# Spatial patterns of dengue cases in Brazil

**DOI:** 10.1371/journal.pone.0180715

**Published:** 2017-07-17

**Authors:** Fernando Jose Antonio, Andreia Silva Itami, Sergio de Picoli, Jorge Juarez Vieira Teixeira, Renio dos Santos Mendes

**Affiliations:** 1 Departamento Acadêmico de Ciências da Natureza, Universidade Tecnológica Federal do Paraná – Cornélio Procópio, Brazil; 2 Departamento de Física, Universidade Estadual de Maringá, Maringá, Brazil; 3 National Institute of Science and Technology for Complex Systems, Maringá, Brazil; 4 Departamento de Análises Clínicas e Biomedicina, Universidade Estadual de Maringá, Maringá, Brazil; Fundacao Oswaldo Cruz, BRAZIL

## Abstract

Dengue infection plays a central role in our society, since it is the most prevalent vector-borne viral disease affecting humans. We statistically investigated patterns concerning the spatial spreading of dengue epidemics in Brazil, as well as their temporal evolution in all Brazilian municipalities for a period of 12 years. We showed that the distributions of cases in municipalities follow power laws persistent in time and that the infection scales linearly with the population of the municipalities. We also found that the average number of dengue cases does not have a clear dependence on the longitudinal position of municipalities. On the other hand, we found that the average distribution of cases varies with the latitudinal position of municipalities, displaying an almost constant growth from high latitudes until reaching the Tropic of Capricorn leveling to a plateau closer to the Equator. We also characterized the spatial correlation of the number of dengue cases between pairs of municipalities, where our results showed that the spatial correlation function decays with the increase of distance between municipalities, following a power-law with an exponential cut-off. This regime leads to a typical dengue traveling distance. Finally, we considered modeling this last behaviour within the framework of a Edwards-Wilkinson equation with a fractional derivative on space.

## Introduction

Infectious diseases are still a relevant problem for humans [1]. Dengue viruses are the greatest cause of arboviral disease around the world [2]. Dengue is an acute viral disease characterized by two or more symptoms: fever, headache, retro-orbital pain, muscle and joint pains, rash, nausea and vomit. There are four recognized dengue virus serotypes (dengue virus [DENV] 1, 2, 3, and 4). Infection by one of the serotypes is thought to produce lifelong immunity to that particular one but only a few months immunity to the others [2–5]. Secondary infections are more likely to result in severe infections and Dengue Hemorrhagic Fever (DHF) characterized by fever, thrombocytopenia, increased vascular permeability and hemorrhagic disthesis [6]. DHF is a potentially lethal complication that in severe cases can cause circulatory failure [2]. Cross reactive but non-neutralizing antibodies from a previous infection bind to the new infecting serotype and facilitate virus access into cells, resulting in higher peak viral titres [2, 7, 8]. Current, prevention of dengue must focus on the mosquito because there are no specific therapeutic agents for dengue. In 2016, the State of Paraná (Southern Brazil) launched the first public dengue immunization program in the Americas with a novel tetravalent vaccine [9, 10].

Dengue is endemic in most tropical and subtropical countries (Latin America, South-East Asia and Central Africa), where large dengue outbreaks occur, affecting both large and small cities [11–16]. Estimates revealed that dengue epidemics have increased around 30 times throughout the last 50 years. Around 10^8^ new cases have occurred annually in more than 100 endemic countries, putting more than 40% of the global population at risk (*ca*. 2.5 billion of people) [17]. This century, Brazil has reported more cases of dengue fever than anywhere else in the world [18], more than 7 million up to 2013 [19]. The four dengue virus serotypes have spread throughout Brazil [20]. Many municipalities have climatic conditions which are conducive to the proliferation and vectorial capacity of *A. aegypti* [19]. Dengue was first recorded in the state of São Paulo between 1851 and 1853. It was virtually eliminated after a campaign to eradicate yellow fever, which has the same transmitting agent as dengue [21]. Two unprecedented epidemics occurred in 1998 and 2002, and a wide diffusion occurred between 2005 and 2009 corresponding to an overall nationwide increase in dengue incidence [16, 20, 21]. Studies found in the available literature examined dengue epidemics in Brazil, including SIR-type modeling [1, 22], model for vaccine cost-effectiveness [23], patterns of dengue circulation [24, 25], epidemiology [20], and dengue risk during the football World Cup [19]. There are also some examples of other studies concerning spatial patterns of the dengue disease such as, spatiotemporal modeling of climate-sensitive disease risk [26], modeling tools for dengue risk mapping [27], and spatial correlation with socioeconomic and demographic variables in Brazilian municipalities [28].

Dengue is an arboviral disease whose principal vector is the *Aedes aegypti* mosquito [2, 27], an anthropophilic specie closely associated to human habitats. It prefers urban environments [26], feeding and resting mainly inside buildings [29, 30]. It is an efficient vector, highly susceptible to the dengue virus, feeding preferentially on human blood [2]. In addition, it is hypothesized that vertical transmission (from mother to offspring) in the *Aedes* mosquito population may allow the virus to persist during periods unfavorable for transmission to humans [31, 32]. The limits of geographical distribution of the *Aedes* species seem to be related to temperature [27, 30], which is closely connected with latitude. Temperature is a major extrinsic factor affecting many population parameters of insects [33]. Some studies focus on the effect of temperature on the *A. aegypti* such as its population dynamics [30], life history [33], development of mosquitoes [34] and larval development [35]. In general, the temperature is not related with longitude, which could imply that the dengue incidence does not depend on the longitude if the population were homogeneous. Unlike most mosquitoes, the *A. aegypti* takes more than one blood meal before the eggs are laid and finds habitats for its larvae in water storage containers and domestic rubbish [2]. Humans and mosquitoes are the principal hosts to the dengue virus; the mosquito remains infected throughout its life, but the virus is only known to cause illness in humans [2]. The number of dengue cases in a region is expected to be connected with the concentration of vectors and with the distribution of susceptible and already infected individuals. The diffusion of dengue is the result of a complex process involving the spread of *Aedes* mosquitoes and their adaptation to urban environments, as well as population mobility which facilitates the circulation of the virus [16]. Many other factors like meteorological variables also play a central role in the number of new dengue cases in specific regions that are strongly influenced by the transmission cycle [16, 36–39]. Apart from the dengue virus, the *A. aegypti* is also a vector for other tropical viruses such as yellow fever, chikungunya virus and zika virus [40].

In the previous paragraphs, several studies about dengue were point out, such as those related to the modeling of dengue dynamics [1], spatial correlation [25, 28] and evolution in space and time [22] in specific cities, spreading among of cities joined by route [16], and vulnerability associated to water and climate within a state [24]. In general, investigations were based on data of some small regions. However, it would also be helpful to have studies that focus on large scales, giving us a more embracing analysis. For instance, taking all Brazilian municipalities into account, where the focus of analyses could be sensitive variables, such as their populations, localizations and numbers of dengue cases while trying to fill this gap in available dengue studies, at least in part, we employed these variables to investigate the spatio-temporal dynamics of dengue cases in Brazil using statistical physics tools. This multidisciplinary approach has become usual in many scenarios and it contributes towards a better understanding of such systems, uncovering patterns or laws that rule the dynamics of systems as a whole. Examples of such investigations include the study of physiological signals [41, 42], mortality of animals after accidents [43], population dynamics [44–46] and spreading of diseases [47, 48]. We investigated dengue cases per se, their relationship with the population and their connection with the geographic location of Brazilian municipalities. We concentrated our studies on a period of years with specific attention to the relationship between population and number of dengue cases, dependence on dengue cases as well as the latitude and spatial correlation of dengue cases between pairs of municipalities. Our approach consisted in disclosing statistical patterns and in modelling the findings within a reductionist framework.

## Data

We extracted the data on dengue cases for each Brazilian municipality directly from DATASUS [49], which is a national public health system database freely available online and maintained by the Data processing Department of the Brazilian Health System. Specifically, we obtained the quantity

$$\begin{array}{c} z_{i,t}\;(\text{the}\;\text{number}\;\text{of}\;\text{new}\;\text{dengue}\;\text{cases}\;\text{diagnosed}\;\text{in}\;\text{municipality}\;i\;\text{during}\;\text{the}\;\text{year}\;t). \end{array}$$ (1)

As a whole, we analyzed data of approximately 6 million dengue cases diagnosed between January 2001 and December 2012. We used data of 5069 Brazilian municipalities with at least one dengue case during this period. Cases were confirmed by clinical and epidemiological evidence and with epidemiological investigations carried out by local health surveillance teams. Approximately 30% of dengue cases were laboratory-confirmed. During epidemics, dengue cases were mostly classified by clinical and epidemiological criteria because of limited laboratory capacity [16, 20]. Dengue is often asymptomatic, so the real number of cases can be larger than the data reported. Furthermore, reports can be considerably underestimated, especially during low endemicity periods, due to the differential operation of health services. However, during outbreaks, the number of cases can even be overestimated. Dengue outbreaks often provoke social upheaval, attracting intense media attention [16]. The data related to the population size in each Brazilian municipality was obtained from IBGE, a Brazilian institution that carries out a national census every 10 years. These data are also freely available online [50]. For a more precise approach, we employed data on the population of the municipalities projected for each year without census [51]. The dataset analyzed in this work is attached as supplementary material (see S1 Table).

## Data analysis

### The profile of annual dengue cases

**Fig 1:**
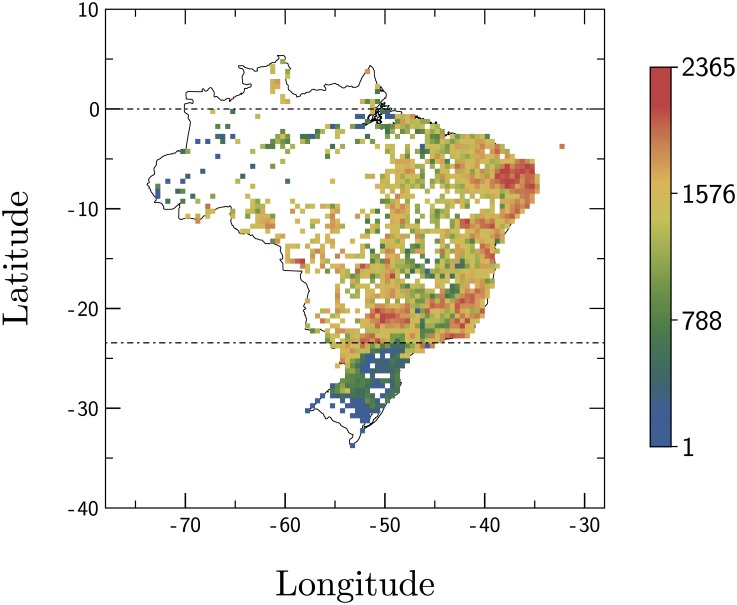
Spatial distribution of dengue cases. Average number of dengue cases per 10^3^ people between 2001 and 2012. Data on the population of the municipalities was projected for each year without census. The color scheme indicates that the major incidence of dengue is found in the extreme Northeastern and Southern Brazilian regions. The dashed lines correspond to the Equator and the Tropic of Capricorn.

The evolution of the average number of dengue cases per 10^3^ people each year is depicted in Fig 1. A general profile of the average number of dengue cases during each year is presented in Supplementary Information as S1 Fig. It is notable from the figure that the extreme Northeast (red and yellow) is the most affected area, followed closely by the Southern regions (yellow).

The population distribution in Brazil is quite irregular, there are clusters on the coastal zones, specially in the Southeast and Northeast. Another important nucleus is the Southern region. The less populated areas are situated in the Mideast and the North [52]. In the Northern region (comprising the largest part of the Amazon forest), there was a low density of cases in spite of the warn and humid climate, favorable to the *A. aegypti* mosquito, this could be explained by the low urban population density [52].

**Fig 2:**
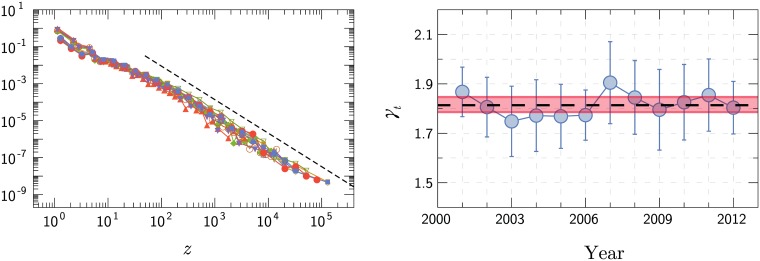
Dengue occurrence in municipalities. (a) Probability Density Function *P*(*z*_*i*,*t*_) for a total of dengue cases per year. The dashed line is a power law with exponent *γ* = 1.81 and serves as a guide to the behaviour of the tail, that includes approximately three decades. (b) Temporal evolution of the exponent *γ*_*t*_ of the power laws for each year between 2001 and 2012. The error bars are 99% confidence intervals, indicating an approximately constant value. The dashed line represents the average value *γ* = 〈*γ*_*t*_〉 = 1.81 and the respective 99% bootstrapped confidence intervals.

Where municipalities are concerned, a pattern emerges: the annual distribution of *z*_*i*,*t*_ follows a power law for large values, *ca*. ∼10^2^. Power laws are usually used to describe a distribution that is heavy-tailed, *i.e*., the tail falls to zero much slower than an exponential function. Power law distributions are surprisingly common in science, they have been observed in a number of different areas, such as in extinction risk due to climate change [53], intensity of wars [54], vertical transmission of culture and family names [55], number of hits on web pages [56], human travel [57], and epidemics among isolated populations [58]. Fig 2A illustrates the distributions of *z*_*i*,*t*_ within municipalities for a fixed number of years (*t* varying from 2001 up to 2012) in comparison to a power law in the form

$$\begin{array}{c} P\left(z_{i,t}\right)\;\propto \;z_{i,t}^{-\gamma }, \end{array}$$ (2)

where *P*(*z*_*i*,*t*_) is the probability of finding *z*_*i*,*t*_ new cases in municipality *i* during the year *t*, with exponent *γ* = 〈*γ*_*t*_〉 = 1.81 ± 0.03 (99% confidence interval). This value is an arithmetic average for the maximum likelihood of fit to the annual power laws 〈*γ*_*t*_〉, shown in Fig 2B. All the power laws are robust for around three decades, that is, from 10^2^ to 10^5^ cases, and the Cramér-von Mises test supports that they can not be rejected at a confidence of 99%. The Cramér-von Mises test is used to judge the goodness of fit of a distribution, employing the cumulative distribution function compared to the given empirical distribution [59–61]. It appears to be more robust in the presence of a few extreme observations compared to other statistical tests [62]. It is notable that, considering the error bars for 99% bootstrapped confidence intervals, the annual *γ*_*t*_ exponents remain systematically constant during the whole period. The power law behavior and its *γ* exponent resemble the values found for AIDS in Brazil *γ* ≃ 1.87 [48]. This might suggest that the spreading of dengue fever has more influence of human circulation than *Aedes*, since a new outbreak marks the arrival of a new serotype (infected person or vector) in the region that had previously presented the arthropod vectors [31].

### Dengue cases *vs* population

**Fig 3:**
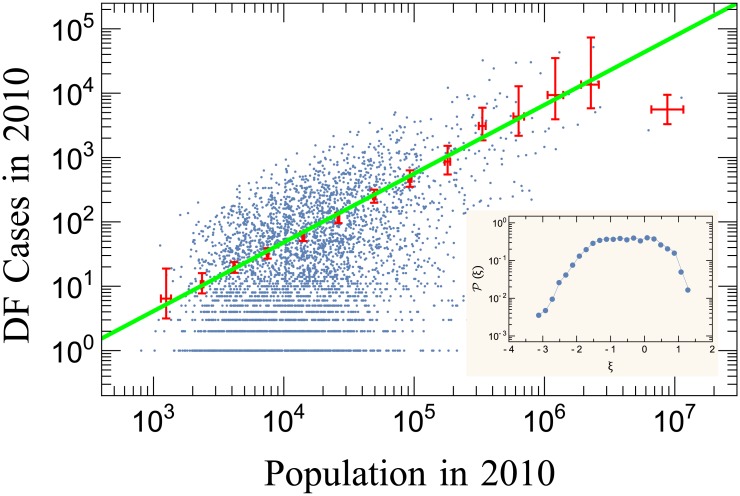
Allometry between dengue cases and population. Each blue dot represents the number of dengue infections in 2010 in a specific municipality versus the population living in that municipality. The red marks represent window average values (15 logarithmically spaced windows chosen through the Wand’s procedure [63]) and the error bars are 99% bootstrapped confidence intervals for these means. The continuous green line is a fit to the window average values. The curve is a power law with exponent *δ* = 1.07 ± 0.02 (99% confidence interval; *R*^2^ = 0.995). The inset shows in log-linear scale the distribution of the residuals around the average tendency described by the spline interpolation.

As pointed out before, the number of new infections (illustrated in Fig 1) depends on many factors, and it is not expected to be constant for different municipalities. Using the Brazilian population data we identified how *z*_*i*,*t*_ scales with population size *p*_*i*,*t*_ in municipality *i* in year *t*. As shown in Fig 3, the curve obtained is well-described by an average scaling law of the type

$$\begin{array}{c} z_{i,t}\;\propto \;p_{i,t}^{\delta }, \end{array}$$ (3)

where *δ* is constant. A linear fit to the windows average values (15 logarithmically spaced windows chosen through Wand’s procedure [63]) provided the exponent value *δ* = 1.07 ± 0.02 (99% confidence interval; *R*^2^ = 0.995)—see also [64]. The result *δ* ≃ 1, in particular, indicates that the average behaviour of *z*_*i*,*t*_ scales almost linearly with the population in the affected region, *i.e*., the number of cases increase with the growth of the population of the municipalities. It should also be noted that this almost isometric scaling law agrees with the one obtained for mortality rates due to influenza and pneumonia in cities of the United States around 1918 [65]. The linear law indicates that dengue has a strong local dependence, different to the usual super-linear behavior which is commonly found in the literature concerning epidemics with social appeal [48]. Also, for many social contexts, such as urban indicators [66, 67], in the case of dengue fever, its relation with socio-economic factors is controversial [28, 68]. A consequence of Eqs 2 and 3 is that

$$\begin{array}{c} P\left(z_{i,t}\right)\;\propto \;p_{i,t}^{-\gamma }, \end{array}$$ (4)

with *γ* ≃ 1.81. This value is larger than other allometric exponents to urban indicators found in the literature with regard to the population, such as unemployment (*γ* = 1.18) and sanitation (*γ* = 1.00) [69].

According to the inset of Fig 3, we found that the distribution of the residuals *ξ* (difference between the data and the average value) resembles a Gaussian, evidencing a random nature intrinsic to the distribution of fluctuations.

### Dengue cases *vs* geographic coordinates of municipalities

**Fig 4:**
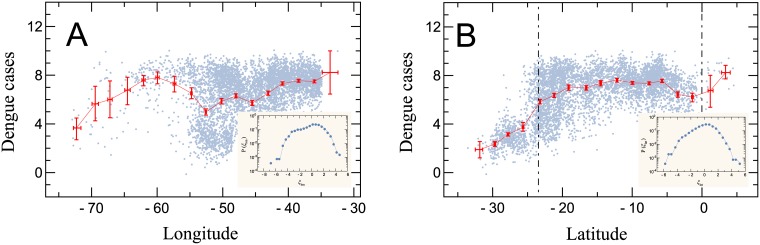
Dependence of dengue cases on the geographical coordinates of municipalities. Average number of dengue cases per million people (in log scale) between 2001 and 2012 as function of the (a) longitudes and (b) latitudes. Each blue dot represents the average of dengue cases per municipality. The red marks are window average values (17 equally spaced windows) and the error bars correspond to 99% bootstrapped confidence intervals for these means. The continuous line is a first order interpolation of the average data. The dashed lines in (b) correspond to the Tropic of Capricorn and the Equator. The bins were chosen through the Wand’s procedure and the inset shows the distribution of the residuals around the average tendency described by the spline interpolation in log-linear scale. We note that the average values of cases per million people fluctuate around a constant value for different longitudes. Nonetheless, another pattern emerges for different latitudes: *(i)* an almost linear growth from high longitudes up to the mid-latitude ∼ −20°; *(ii)* an approximate plateau from the mid-latitude up to the Equator.

Since dengue epidemics mostly affect the tropical region of the globe, it is reasonable to investigate the connection between the number of dengue cases to geographical coordinates. Fig 4 reveals how the average number of dengue cases per million people (in log scale) correlates with the geographic coordinates of the mentioned municipalities. We found that the average values of *z*_*i*,*t*_ fluctuate around a systematically constant value for different longitudes, due to population clusterization in coastal zones (Southeast and Northeast) [52]. Nonetheless, another pattern emerges for *z*_*i*,*t*_ for different latitudes: *(i)* an almost linear growth from high latitudes until the mid-latitude ∼20° S, despite the cluster of population in Southern Brazil; *(ii)* an approximate plateau from the mid-latitude up to the Equator. The pattern (*i*) is consistent with the fact that the southern limit of distribution of the *A. aegypti* in South America, is given in literature by the line joining the city of Tacna in Southern Peru (17°36′ S, 70°12′ W) to the city of Bahía Blanca in Argentina (38°43′ S, 62°16′ W), with a winter isotherm of 10°C [29, 30]. The impact of lower temperatures on species is a slow down of development, generating smaller individuals, decreasing the capacity of attack, and consequently, its efficiency as a vector [29, 30, 33]. Considering that the number of new cases of dengue is related to the density of vectors or their efficiency, our results are within the geographic limits. This implies that the distribution of mosquitoes or their capacity as a vector is not constant throughout the area where they are found, but only in higher latitudes (≳ 20° S) and there is a linear decrease with the decrease of latitude (∼20° S to ∼30° S).

The distribution of the residuals (*ξ*_*lon*_ and *ξ*_*lat*_) resemble a Gaussian. They do not obey any form of common distribution and are also different to the residuals obtained from the allometric scaling. Their shape is mainly influenced by the geographical distribution of the municipalities.

### Spatial correlation of the incidence rate of dengue cases

**Fig 5:**
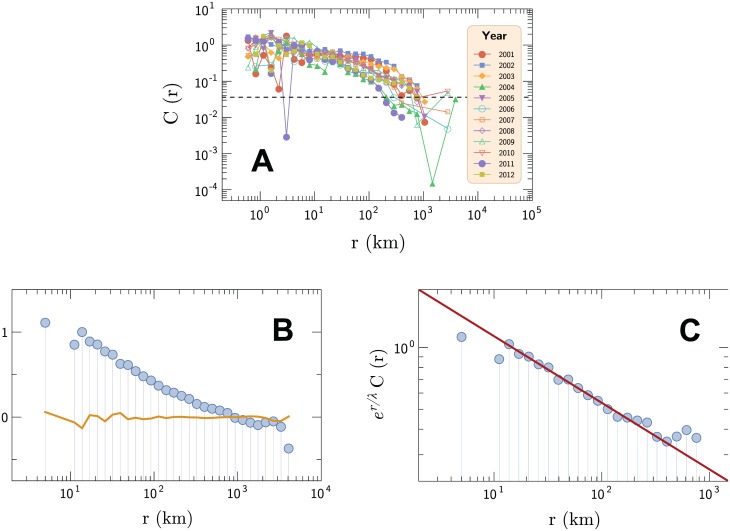
Decay of the spatial correlations of dengue cases. (a) Temporal evolution from 2001 up to 2012 of the spatial correlation shown in log-log scale for the annual number of incidence rates among Brazilian municipalities. The dashed black line is a guide to distinguish from random noise with a confidence level of 99%. (b) Annual average of the spatial correlation shown in log-linear scale for the total number of dengue cases among municipalities between 2001 and 2012. The continuous red line is a non-linear fit of Eq (6) to the data; the parameters where found to be *c*_0_ = 2.40 ± 0.24, *θ* = 0.32 ± 0.03 and *λ* = 375 ± 66 km, errors which represent a 99% confidence interval. The dashed black line is a guide to distinguish from random noise with a confidence level of 99%, while the continuous orange line is the output of the spatial correlation function computed after shuffling the position of the municipalities, showing the lack of spatial correlations. (c) Plot in log-log scale, showing the power law component of the spatial correlation through the data re-escalated by multiplying an exponential factor.

We conclude our study about dengue by focusing on the existence of spatial correlation in *z*_*i*,*t*_. To investigate this aspect, we evaluated the spatial correlation function of *z*_*i*,*t*_ between pairs of municipalities that are *r* kilometers apart. The spatial correlation of some indicators can be originated by the natural distribution of the population in the municipalities. Aiming to remove this effect and considering that *δ* ≃ 1, we actually used the quantity *y*_*i*,*t*_ = *z*_*i*,*t*_/*p*_*i*,*t*_, *i.e*., the incidence rate in municipality *i* in year *t*. Specifically, we computed

$$\begin{array}{c} C\left(r\right)=\frac{\left\langle \left[y_{i,t}-\mu \left(r\right)\right]\left[y_{j,t}-\mu \left(r\right)\right]\right\rangle {|}_{r_{i,j}=r}}{{\sigma (r)}^{2}}\;, \end{array}$$ (5)

where *μ*(*r*) represents the mean value, *σ*(*r*) denotes the standard deviation of the quantity *y*_*i*,*t*_ of municipalities separated by *r* kilometers and 〈…〉|_*r*_*i*,*j*_ = *r*_ stands for the arithmetic average over municipalities whose distance *r*_*i*,*j*_ is equal to *r*. To proceed with the analysis, we considered logarithmically spaced intervals of *r* for evaluating Eq (5). Fig 5A shows the temporal evolution of the spatial correlation *C*(*r*) in log-log scale for the annual average number of dengue cases among municipalities. There is a remarkably quick decay of the correlation function. In addition, this behavior can be distinguished from random noise for between two to three decades.

Considering the annual arithmetic average incidence rate per municipality over a period of 12 years (*i.e*., replacing *y*_*i*,*t*_ by $\langle y_{i}\rangle =\frac{1}{12}\sum_{t=1}^{12}y_{i,t}$ in Eq (5)), we identified that *C*(*r*) follows a power law with an exponential cut-off, as shown in Fig 5B. In general, the curve we obtained is well described by

$$\begin{array}{c} C\left(r\right)=c_{0}\;r^{-\theta }\;e^{-r/\lambda }\;, \end{array}$$ (6)

where *c*_0_ is a scale factor, *θ* is a critical exponent and *λ* is the correlation length. This type of curve is a typical result in the neighborhood of critical points [70]. In our case, a non-linear fit to the data (see the continuous red line on Fig 5B) leads to *c*_0_ = 2.40 ± 0.24, *θ* = 0.32 ± 0.03 and *λ* = 375 ± 66 km, whose errors represent a 99% confidence interval. Based on the Cramér-von Mises test, these values can not be rejected at a 99% level of confidence (*p*-value 0.571). Confirming our results, the spatial correlation present in the data is destroyed and can not be distinguished from random noise with a simple shuffling of data. This is illustrated by the continuous orange line (shuffled) and the dashed black line (threshold of random noise) in Fig 5B.

The correlation length, *λ* = 375 km, represents an average distance over which the intensity of the number of dengue cases persists. The mobility of *A. aegypti* is only a few meters, flying long distances only in exceptional circumstances, for example, it is capable of a sustained flight for 1 kilometer over water [29]. As such, it is reasonable to assume that the virus dispersal over long distances is made either through the circulation of infected individuals or by carrying infected mosquitoes. Our study provides another view about space correlations, since previous investigations focused on micro regions [26, 28] and special cases of road networks connecting medium sized municipalities to small ones [16]. Furthermore, in a similar study, although related to spread obesity, it was pointed out that parameter *λ* is typically connected to the finitude of the empirical data, depending sub-linearly on the size of the system [71]. Fig 5C shows the critical exponent *θ* of the power law decay, considering *C*(*r*) ∝ *e*^ − *r*/*λ*^.

A correlation function decaying as power law can be found in several studies, such as in literary texts [72]. The average incidence rate is long-range correlated with respect to space, and the correlation function *C*(*r*) decays even faster than a power law due to the finitude of the system. Reinforcing this result is that, by shuffling the average dengue rates among municipalities, the spatial correlations are destroyed and the profile of the residual distributions (insets of the Fig 4A and 4B) is drastically changed. Specifically, both the residual distributions for latitude and longitude become approximately equal, simply reflecting the spatial distribution of Brazilian municipalities.

As previously pointed out, the spread and growth of dengue is related to mobility. In modeling the correlation *C*(*r*) with respect to spatial correlation of human mobility, we employed the Edwards-Wilkinson equation [73]. This equation initially intended to investigate the growth of interfaces [73, 74], but it may be useful to investigate a diffusive process which starts at the origin and grows radially, intermingled with a random noise *η* (random source). The Edwards-Wilkinson equation was used in the study of other collective phenomena such as elections [75], homicide crimes [76] and lightning activity [77]. Unfortunately, this description is not a sufficient approximation to describe the spreading of an epidemic such as dengue. One notable reason is the small world feature [78] due to long trips by infected people [15]. As such, it becomes natural to generalize the Edwards-Wilkinson equation by replacing the Brownian motion so as to incorporate long jumps whose lengths could follow. Therefore, we used a Lévy distribution, because such characteristics make our model more consistent to human mobility. In fact, in contrast with the Gaussian cases, Lévy flights are characterized by frequent movement for short distances, interrupted by random changes of direction that are only occasionally followed by movements over longer distances. Sorting out the displacement lengths covered in these journeys by size frequency, results in a distribution with a long tail that obeys a power law [79]. This pattern, for instance, has been found in the displacement of animals [80, 81], and humans in local [82], nation-wide [57] and global sphere [79]. Thus, the model under consideration here is written as a generalized Edwards-Wilkinson equation:

$$\begin{array}{c} \frac{\partial }{\partial t}\;y\left(r,t\right)=\nu \;\nabla^{\alpha }\;y\left(r,t\right)+\eta \left(r,t\right)\;, \end{array}$$ (7)

where *y*(**r**, *t*) represents the average incidence rate in a municipality localized by the position of vector **r** in relation to another municipality forming a pair, *ν* is a diffusion constant, *η*(**r**, *t*) is a random noise with zero mean and finite variance uncorrelated in space and time. ∇^*α*^ is a Riesz fractional operator and leads to long jumps, playing the role of a Laplacian arbitrary order, defined by $F\left\{\nabla^{\alpha }f\left(r\right)\right\}=-k^{\alpha }F\left\{f\left(r\right)\right\}$, where *k* = |**k**| and F{⋯} is the Fourier transform [83, 84]. Usually, the range 0 < *α* ≤ 2 is used to assure that *y*(**r**, *t*) is non-negative when *η*(**r**, *t*) = 0. Notice also that a smaller *α* favors longer jumps. Furthermore, the limit *α* = 2 corresponds to the usual Laplacian, leading again to the Edwards-Wilkinson equation and consequently to a Gaussian distribution.

In the deterministic case (*i.e*., when *η*(**r**, *t*) = 0), the solution of Eq (7) in the infinity space can be obtained analytically by making use of a Lévy distribution [85]. In general, it can be demonstrated that for an infinity system, Eq (7) leads to a height-height correlation function in the form of

$$\begin{array}{c} C\left(r,t\right)∼r^{-(d-\alpha )}\;, \end{array}$$ (8)

where *d* represents the spatial dimension of the system analyzed [86, 87]. Because humans and *Aedes* mosquitoes live essentially close to the Earth’s surface, we do not use data on altitude. As a consequence, *d* = 2. Our model does not incorporate data from local relief features, that is, altitude is not an independent variable in this study. As such, our model only used the average behavior as regards the altitude. Thus, the use of the previous *θ*, *θ* = 0.32 ± 0.03, in the relation *θ* = *d* − *α* leads to *α* ≃ 1.68. This result shows the consistency of our model in the spread of dengue with the possibility of long jumps, in contrast with the case *α* = 2, where only short-ranged jumps occur. Additionally, even when long range correlations are known to be present, a cut-off value will eventually emerge. In general, as previously pointed out, this kind of cut-off may be associated with the finitude of the system; an example related to humans occurs in the study of obesity prevalence [71]. The incorporation of this aspect in our study was obtained by multiplying the power law of our model by an exponential cut-off.

## Conclusion

In this work, we found patterns in dengue cases for Brazilian municipalities: the distribution of dengue fever cases follows a power law; the relationship between the population and the number of cases obeys an isometric rule; the existence of a dependence on the number of dengue cases with the latitude of the municipalities; a spatial correlation decaying as a power law with an exponential cut-off.

With small spatial scales (*i.e*., at municipal level), the distribution of dengue cases showed a robust power law behavior for twelve years with an average negative exponent, as illustrated in Fig 2. A similar result was found to AIDS in Brazil [48]. In the AIDS case, humans are the main carrier of the disease. The fact that dengue presents a similar distribution could be an indication that human mobility is more consequential than vector mobility, which has a limited locomotion capacity. Furthermore, the vector can be transported by population movement. The appearance of the power law behavior is a distinct pattern: power laws decay slower than exponential regimes and are ubiquitous in nature, commonly emerging from critical phenomena.

We also analyzed how the distribution of dengue cases scales with the population of the region where they were centered. In relation to the population of the municipalities, Fig 3 illustrates the almost isometric rule between the number of dengue cases against the population of the municipalities. Population size did not affect dengue case rates significantly in Brazilian municipalities in the period 2001–2012. Similar behavior has been identified for other illnesses, for instance, in the study of mortality rates by influenza and pneumonia for cities in the United States around 1918 [65].

In Fig 4, we characterized dengue epidemics encompassing all Brazilian municipalities by taking their geographic location into account. We found that dengue cases have an approximately constant tendency in their average values for a wide range of longitudes. As for the dependence of dengue cases on the latitude of municipalities, two distinct regions were identified: an almost linear growth up to the Tropic of Capricorn and an approximate plateau between the Tropic of Capricorn and the Equator. Considering that the number of new dengue cases is related to the density of vectors, our results are within the limits found in the literature concerning the *A. aegypti*. More precisely, such results imply that the distribution of mosquitoes is not constant in all areas where it is found, but only in higher latitudes where there is a linear decrease with the increase of latitude.

We conclude our study with an investigation of the spatial correlations of dengue cases between pairs of municipalities (Fig 5). Our results show that the correlation function in terms of the distance between each pair of municipalities decays as a power law with an exponential cut-off. We proposed to model the power law decay via a generalization of the Edwards-Wilkinson equation with a fractional derivative on space. This model connects the spatial correlation of the number of new dengue cases diagnosed per capita among municipalities with human mobility in the transmission of dengue. In this context, the correlation length represents an average distance over which the intensity of dengue cases persists. Our results could be more embracing than those found in literature, where spatial correlation is studied in microregions or only for a few special cases. Our findings indicate that population mobility has an important role in the spread of the dengue virus [16, 29, 31]. Although there is a lack of studies available about human mobility in Brazil, our results imply that the risk area brought about by a large outbreak could be larger than the one commonly considered in local studies.

Presently, Brazil faces a complex epidemiological scenario characterized by simultaneous circulation of three arboviruses—dengue, chikungunya and zika –, transmitted by the *Aedes Aegypti* mosquito. Knowing more about the spread of dengue fever can also intensify the fight against these other infections. Arboviruses are a huge concern to public health due to a diversity of infectious agents involved, unexpected clinical manifestations and potential complications associated with the cases. Furthermore, there is difficulty in the implementation and maintenance of educational and sanitary measures. Any additional information about spreading patterns of these diseases may be useful in the preparation and guidance of states and municipalities towards the adoption of measures for vector control and epidemiological surveillance. For example, if a municipality presents an amount of cases higher than was identified in the average allometric behaviour, the incidence of dengue should be given special attention by public health organizations. The region between ∼ −20° and the equator had a higher incidence of dengue and, therefore, this region should receive greater attention from public authorities. The distance of correlation indicates that efforts against the spread of dengue should not be local, but cover regions in accordance with this correlation.

In order to improve our understanding of the spatio-temporal dynamics of dengue, it would be helpful if dengue cases in other regions as large as Brazil were also investigated. In particular, to verify if the dynamics explored in our study could serve as a model for investigation in regions with similar climate. More specifically, how the peculiarities inherent to each region can cause interference in the patterns obtained in our analysis. Thus the possible final results, obtained with a large database, could be seen as a basis for public health policies such as those exemplified in the previous paragraph.

## Supporting information

S1 Table Dataset of dengue cases in Brazillian municipalities between 2001 and 2012. Data of 5069 Brazilian municipalities with at least one dengue case during this period.
(CSV)

S1 Fig Spatial distribution of dengue cases during each year. A general profile of the average number of dengue cases from 2001 up to 2012. Data on the population of the municipalities was projected for each year without census. The color scheme indicates that the major incidence of dengue is found in the extreme Northeastern and Southern Brazilian regions. The dashed lines correspond to the Equator and the Tropic of Capricorn.
(PDF)

## References

[1] Pinho STR, Ferreira CP, Esteva L, Barreto FR, Morato e Silva VC, Teixeira MGL. Modelling the dynamics of dengue real epidemics. Philosophical Transactions of the Royal Society A: Mathematical, Physical and Engineering Sciences. 2010;368(1933):5679–5693. 10.1098/rsta.2010.0278 21078642

[2] Gibbons RV, Vaughn DW. Dengue: an escalating problem. BMJ: British Medical Journal. 2002;324(7353):1563 10.1136/bmj.324.7353.1563 12089096 PMC1123504

[3] Monath TP. Dengue: the risk to developed and developing countries. Proceedings of the National Academy of Sciences of the United States of America. 1994;91(7):2395–2400. 10.1073/pnas.91.7.2395 8146129 PMC43378

[4] World Health Organization. Dengue haemorrhagic fever: diagnosis, treatment, prevention and control. Geneva: WHO; 1997.

[5] Gubler DJ. Dengue and dengue hemorrhagic fever. Clinical microbiology reviews. 1998;11(3):480–496. 9665979 10.1128/cmr.11.3.480 PMC88892

[6] Burke DS, Nisalak A, Johnson DE, Robert McN Scott RM. A prospective study of dengue infections in Bangkok. The American Journal of Tropical Medicine and Hygiene. 1988;38(1):172–180. 10.4269/ajtmh.1988.38.172 3341519

[7] Thein S, Aung MM, Shwe TN, Aye M, Zaw A, Aye K, et al Risk factors in dengue shock syndrome. The American Journal of Tropical Medicine and Hygiene. 1997;56(5):566–572. 10.4269/ajtmh.1997.56.566 9180609

[8] Vaughn DW, Green S, Kalayanarooj S, Innis BL, Nimmannitya S, Suntayakorn S, et al Dengue viremia titer, antibody response pattern, and virus serotype correlate with disease severity. Journal of Infectious Diseases. 2000;181(1):2–9. 10.1086/315215 10608744

[9] Capeding MR, Tran NH, Hadinegoro SRS, Ismail HIHM, Chotpitayasunondh T, Chua MN, et al Clinical efficacy and safety of a novel tetravalent dengue vaccine in healthy children in Asia: a phase 3, randomised, observer-masked, placebo-controlled trial. The Lancet. 2014;384(9951):1358–1365. 10.1016/S0140-6736(14)61060-6 25018116

[10] Sanofi Pasteur | Dengue Info;. http://www.dengue.info/#overlay=content/brazil-vaccination-starts.

[11] Cummings DAT, Irizarry RA, Huang NE, Endy TP, Nisalak A, Ungchusak K, et al Travelling waves in the occurrence of dengue haemorrhagic fever in Thailand. Nature. 2004;427:344–347. 10.1038/nature02225 14737166

[12] Guzman A, Istúriz RE. Update on the global spread of dengue. International Journal of Antimicrobial Agents. 2010;36S:S40 10.1016/j.ijantimicag.2010.06.018 20833000

[13] Gharbi M, Quenel P, Gustave J, Cassadou S, Guy La Ruche LG, Marrama L. Time series analysis of dengue incidence in Guadeloupe, French West Indies: Forecasting models using climate variables as predictors. BMC Infectious Diseases. 2011;11:166 10.1186/1471-2334-11-166 21658238 PMC3128053

[14] Hu W, Clement A, Williams G, Tong S. Spatial analysis of notified dengue fever infections. Epidemiology and Infection. 2011;139:391–399. 10.1017/S0950268810000713 20392302

[15] Wilder-Smith A. Dengue infections in travellers. Paediatrics and International Child Health. 2012;32(s1):28–32. 10.1179/2046904712Z.00000000050 22668447 PMC3381444

[16] Barcellos C, Lowe R. Expansion of the dengue transmission area in Brazil: the role of climate and cities. Tropical Medicine & International Health. 2014;19(2):159–168. 10.1111/tmi.12227 24286460

[17] WHO | Dengue;. http://www.who.int/denguecontrol/en/.

[18] Teixeira MG, Costa MdCN, Barreto F, Barreto ML. Dengue: twenty-five years since reemergence in Brazil. Cadernos de Saúde Pública. 2009;25:S7–S18. 10.1590/S0102-311X2009001300002 19287868

[19] Lowe R, Barcellos C, Coelho CA, Bailey TC, Coelho GE, Graham R, et al Dengue outlook for the World Cup in Brazil: an early warning model framework driven by real-time seasonal climate forecasts. The Lancet Infectious Diseases. 2014;14(7):619–626. 10.1016/S1473-3099(14)70781-9 24841859

[20] Siqueira JB Jr, Martelli CMT, Coelho GE, da Rocha Simplício AC, Hatch DL. Dengue and dengue hemorrhagic fever, Brazil, 1981–2002. Emerging infectious diseases. 2005;11(1):48 10.3201/eid1101.031091 15705322 PMC3294356

[21] Saba H, Miranda JGV, Moret MA. Self-organized critical phenomenon as a *q*-exponential decay—Avalanche epidemiology of dengue. Physica A: Statistical Mechanics and its Applications. 2014;413:205–211.

[22] Silveira GP, de Barros LC. Numerical methods integrated with fuzzy logic and stochastic method for solving PDEs: An application to dengue. Fuzzy Sets and Systems. 2013;225(0):39–57. 10.1016/j.fss.2013.04.003

[23] Durham DP, Mbah MLN, Medlock J, Luz PM, Meyers LA, Paltiel AD, et al Dengue dynamics and vaccine cost-effectiveness in Brazil. Vaccine. 2013;3(37):3957–3961. 10.1016/j.vaccine.2013.06.036 PMC3755607 23791696

[24] Dickin SK, Schuster-Wallace CJ. Assessing changing vulnerability to dengue in northeastern Brazil using a water-associated disease index approach. Global Environmental Change. 2014;29(0):155–164. 10.1016/j.gloenvcha.2014.09.007

[25] Nogueira M, Colombo T, Vedovello D, Mondini A, Drumond B, Favaro E. Dengue virus surveillance in a medium size city in Brazil reveals a complex pattern of serotypes and strains circulation. International Journal of Infectious Diseases. 2014;21, Supplement 1(0):20 10.1016/j.ijid.2014.03.452

[26] Lowe R, Bailey TC, Stephenson DB, Graham RJ, Coelho CAS, Carvalho S M, et al Spatio-temporal modelling of climate-sensitive disease risk: Towards an early warning system for dengue in Brazil. Computers & Geosciences. 2011;37(3):371–381. 10.1016/j.cageo.2010.01.008

[27] Louis VR, Phalkey R, Horstick O, Ratanawong P, Wilder-Smith A, Tozan Y, et al Modeling tools for dengue risk mapping-a systematic review. International journal of health geographics. 2014;13(1):50 10.1186/1476-072X-13-50 25487167 PMC4273492

[28] Mondini A, Chiaravalloti-Neto F. Spatial correlation of incidence of dengue with socioeconomic, demographic and environmental variables in a Brazilian city. Science of The Total Environment. 2008;393(2–3):241–248. 10.1016/j.scitotenv.2008.01.010 18262225

[29] Christophers SSR. *Aëdes aegypti* (L.) The Yellow Fever Mosquito. Cambridge: Cambridge University Press; 1960.

[30] Simoy MI, Simoy MV, Canziani GA. The effect of temperature on the population dynamics of Aedes aegypti. Ecological Modelling. 2015;314:100–110. 10.1016/j.ecolmodel.2015.07.007

[31] Gubler DJ. Arboviruses as imported disease agents: the need for increased awareness In: Schwarz TF, Siegl G, editors. Imported Virus Infections. Vienna: Springer Vienna; 1996 p. 21–32. 10.1007/978-3-7091-7482-1_3 8800801

[32] Adams B, Boots M. How important is vertical transmission in mosquitoes for the persistence of dengue? Insights from a mathematical model. Epidemics. 2010;2(1):1–10. 10.1016/j.epidem.2010.01.001 21352772

[33] Grech MG, Sartor PD, Almirón WR, Luduea-Almeida FF. Effect of temperature on life history traits during immature development of Aedes aegypti and Culex quinquefasciatus (Diptera: Culicidae) from Córdoba city, Argentina. Acta Tropica. 2015;146:1–6. 10.1016/j.actatropica.2015.02.010 25733491

[34] Mohammed A, Chadee DD. Effects of different temperature regimens on the development of Aedes aegypti (L.) (Diptera: Culicidae) mosquitoes. Acta Tropica. 2011;119(1):38–43. 10.1016/j.actatropica.2011.04.004 21549680

[35] Ledesma N, Harrington L. Fine-scale temperature fluctuation and modulation of Dirofilaria immitis larval development in Aedes aegypti. Veterinary Parasitology. 2015;209(1–2):93–100. 10.1016/j.vetpar.2015.02.003 25747489 PMC4390526

[36] Patz J, Epstein P, Burke T, Balbus J. Global climate change and emerging infectious diseases. Journal of the American Medical Association. 1996;275(3):217–223. 10.1001/jama.275.3.217 8604175

[37] Hsieh YH, Chen CWS. Turning points, reproduction number, and impact of climatological events for multi-wave dengue outbreaks. Tropical Medicine and International Health. 2009;14(6):628–38. 10.1111/j.1365-3156.2009.02277.x 19392743

[38] Shang CS, Fang CT, Liu CM, Wen TH, Tsai KH, King CC. The Role of Imported Cases and Favorable Meteorological Conditions in the Onset of Dengue Epidemics. PLoS Neglected Tropical Diseases. 2010;4(8):e775 10.1371/journal.pntd.0000775 20689820 PMC2914757

[39] Hii YL, Zhu H, Ng N, Ng LC, Rockl’ov J. Forecast of Dengue Incidence Using Temperature and Rainfall. PLoS Neglected Tropical Diseases. 2012;6(11):e1908 10.1371/journal.pntd.0001908 23209852 PMC3510154

[40] Portal da Saúde;. http://portalsaude.saude.gov.br/images/pdf/2015/agosto/26/2015-020-publica----o.pdf.

[41] Ivanov PC, Chen Z, Hu K, Stanley HE. Multiscale aspects of cardiac control. Physica A: Statistical Mechanics and its Applications. 2004;344(3–4):685–704. 10.1016/j.physa.2004.08.016

[42] Bartsch R, Kantelhardt JW, Penzel T, Havlin S. Experimental Evidence for Phase Synchronization Transitions in the Human Cardiorespiratory System. Physical Review Letters. 2007;98:054102 10.1103/PhysRevLett.98.054102 17358862

[43] Antonio FJ, Mendes RS, Thomaz SM. Identifying and modeling patterns of tetrapod vertebrate mortality rates in the Gulf of Mexico oil spill. Aquatic Toxicology. 2011;105(1–2):177–179. 10.1016/j.aquatox.2011.05.022 21718661

[44] Milne BT. Motivation and Benefits of Complex Systems Approaches in Ecology. Ecosystems. 1998;1(5):449–456. 10.1007/s100219900040

[45] Yamasaki K, Matia K, Buldyrev SV, Fu D, Pammolli F, Riccaboni M, et al Preferential attachment and growth dynamics in complex systems. Physical Review E. 2006;74:035103 10.1103/PhysRevE.74.035103 17025688

[46] Martinez AS, González RS, Espíndola AL. Generalized exponential function and discrete growth models. Physica A: Statistical Mechanics and its Applications. 2009;388(14):2922–2930. 10.1016/j.physa.2009.03.035

[47] Picoli Sd Junior, Teixeira JJV, Ribeiro HV, Malacarne LC, Santos RPBd, Mendes RdS. Spreading Patterns of the Influenza A (H1N1) Pandemic. PLoS ONE. 2011;6(3):e17823 10.1371/journal.pone.0017823 21483857 PMC3069037

[48] Antonio FJ, Picoli S, Teixeira JJV, Mendes RS. Growth Patterns and Scaling Laws Governing AIDS Epidemic in Brazilian Cities. PLoS ONE. 2014;9(10):e111015 10.1371/journal.pone.0111015 25340796 PMC4207789

[49] DATASUS;. http://www2.datasus.gov.br/DATASUS/index.php?area=0203.

[50] IBGE;. ftp://ftp.ibge.gov.br/Censos.

[51] IBGE;. ftp://ftp.ibge.gov.br/Estimativas_de_Populacao.

[52] SIDRA;. http://www.sidra.ibge.gov.br/.

[53] Thomas CD, Cameron A, Green RE, Bakkenes M, Beaumont LJ, Collingham YC, et al Extinction risk from climate change. Nature. 2004;427:145–148. 10.1038/nature02121 14712274

[54] Roberts DC, Turcotte DL. Fractality and Self-Organized Criticality of Wars. Fractals. 1998;06:351–357. 10.1142/S0218348X98000407

[55] Zanette DH, Manrubia SC. Vertical transmission of culture and the distribution of family names. Physica A: Statistical Mechanics and its Applications. 2001;295(1):1–8. 10.1016/S0378-4371(01)00046-2

[56] Adamic LA, Huberman BA. Power-law distribution of the world wide web. Science. 2000;287(5461):2115–2115. 10.1126/science.287.5461.2115a

[57] Brockmann D, Hufnagel L, Geisel T. The scaling laws of human travel. Nature. 2006;439 10.1038/nature04292 16437114

[58] Rhodes CJ, Anderson RM. Power laws governing epidemics in isolated populations. Nature. 1996;381:600–602. 10.1038/381600a0 8637594

[59] Cramr H. On the composition of elementary errors: First paper: Mathematical deductions. Scandinavian Actuarial Journal. 1928;1928(1):13–74. 10.1080/03461238.1928.10416862

[60] von Mises R. Wahrscheinlichkeit Statistik und Wahrheit. Berlin, Heidelberg: Springer Berlin Heidelberg; 1936 Available from: http://link.springer.com/10.1007/978-3-662-41863-5.

[61] Stephens MA. EDF Statistics for Goodness of Fit and Some Comparisons. Journal of the American Statistical Association. 1974;69(347):730–737. 10.1080/01621459.1974.10480196

[62] Syrjala SE. A Statistical Test for a Difference between the Spatial Distributions of Two Populations: Ecological Archives E077-001. Ecology. 1996;77(1):75–80. 10.2307/2265656

[63] Wand MP. Data-Based Choice of Histogram Bin Width. The American Statistician. 1997;51(1):59–64. 10.2307/2684697

[64] Rocha LEC, Thorson AE, Lambiotte R. The Non-linear Health Consequences of Living in Larger Cities. Journal of Urban Health. 2015;92(5):785–799. 10.1007/s11524-015-9976-x 26245466 PMC4608943

[65] Acuna-Soto R, Viboud C, Chowell G. Influenza and Pneumonia Mortality in 66 Large Cities in the United States in Years Surrounding the 1918 Pandemic. PLoS ONE. 2011;6(8):e23467 10.1371/journal.pone.0023467 21886792 PMC3158768

[66] Bettencourt LMA, Lobo J, Helbing D, K’uhnert C, West GB. Growth, innovation, scaling, and the pace of life in cities. Proceedings of the National Academy of Sciences of the United States of America. 2007;104(17):7301–7306. 10.1073/pnas.0610172104 17438298 PMC1852329

[67] Alves LGA, Ribeiro HV, Lenzi EK, Mendes RS. Distance to the Scaling Law: A Useful Approach for Unveiling Relationships between Crime and Urban Metrics. PLoS ONE. 2013;8(8):e69580 10.1371/journal.pone.0069580 23940525 PMC3734155

[68] Almeida AS, Medronho RA, Valencia LIO. Spatial analysis of dengue and the socioeconomic context of the city of Rio de Janeiro (Southeastern Brazil). Physica A: Statistical Mechanics and its Applications. 2009;43(4):666–673. 10.1590/s0034-89102009000400013 19649472

[69] Alves LGA, Ribeiro HV, Lenzi EK, Mendes RS. Empirical analysis on the connection between power-law distribution and allometries for urban indicators. Physica A: Statistical Mechanics and its Applications. 2014;409:175–183. 10.1016/j.physa.2014.04.046

[70] Fratini M, Poccia N, Ricci A, Campi G, Burghammer M, Aeppli G, et al Scale-free structural organization of oxygen interstitials in La2CuO4+y. Nature. 2010;466(7308):841–844. 10.1038/nature09260 20703301

[71] Gallos LK, Barttfeld P, Havlin S, Sigman M, Makse HA. Collective behavior in the spatial spreading of obesity. Scientific Reports. 2012;2(454):1–9. 10.1038/srep00454 PMC3400682 22822425

[72] Altmann EG, Cristadoro G, Esposti MD. On the origin of long-range correlations in texts. Proceedings of the National Academy of Sciences of the United States of America. 2012;109(29):11582–11587. 10.1073/pnas.1117723109 22753514 PMC3406867

[73] Edwards SF, Wilkinson DR. The Surface Statistics of a Granular Aggregate. Proceedings of the Royal Society of London Series A: Mathematical and physical sciences. 1982;381(1780):17–31. 10.1098/rspa.1982.0056

[74] Chame A, Reis FDAA. Scaling of local interface width of statistical growth models. Surface Science. 2004;553(1–3):145–154. 10.1016/j.susc.2004.01.048

[75] Borghesi C, Raynal JC, Bouchaud JP. Election Turnout Statistics in Many Countries: Similarities, Differences, and a Diffusive Field Model for Decision-Making. PLoS ONE. 2012;7(5):e36289 10.1371/journal.pone.0036289 22615762 PMC3354000

[76] Alves LGA, Lenzi EK, Mendes RS, Ribeiro HV. Spatial correlations, clustering and percolation-like transitions in homicide crimes. EPL (Europhysics Letters). 2015;111(1):18002 10.1209/0295-5075/111/18002

[77] Ribeiro HV, Antonio FJ, Alves LGA, Lenzi EK, Mendes RS. Long-range spatial correlations and fluctuation statistics of lightning activity rates in Brazil. Europhysics Letters. 2013;104(6):69001 10.1209/0295-5075/104/69001

[78] Albert R, Barabási AL. Statistical mechanics of complex networks. Reviews of modern physics. 2002;74(1):47 10.1103/RevModPhys.74.47

[79] Deutschmann E. The spatial structure of transnational human activity. Social Science Research. 2016;59:120–136. 10.1016/j.ssresearch.2016.04.008 27480376

[80] Viswanathan GM, Afanasyev V, Buldyrev SV, Murphy EJ, Prince PA, Stanley HE. Lévy flight search patterns of wandering albatrosses. Nature. 1996;381(6581):413–415. 10.1038/381413a0 17960243

[81] Sims DW, Southall EJ, Humphries NE, Hays GC, Bradshaw CJA, Pitchford JW, et al Scaling laws of marine predator search behaviour. Nature. 2008;451(7182):1098–1102. 10.1038/nature06518 18305542

[82] Papandrea M, Jahromi KK, Zignani M, Gaito S, Giordano S, Rossi GP. On the properties of human mobility. Computer Communications. 2016;87:19–36. 10.1016/j.comcom.2016.03.022

[83] Compte A. Stochastic foundations of fractional dynamics. Physical Review E. 1996;53:4191–4193. 10.1103/PhysRevE.53.4191 9964735

[84] Anteneodo C, Dias JC, Mendes RS. Long-time behavior of spreading solutions of Schr odinger and diffusion equations. Physical Review E. 2006;73:051105 10.1103/PhysRevE.73.051105 16802916

[85] Metzler R, Klafter J. The random walk’s guide to anomalous diffusion: A fractional dynamics approach. Physics Reports. 2000;339(1):1–77. 10.1016/S0370-1573(00)00070-3

[86] Nattermann T, Tang LH. Kinetic surface roughening. I. The Kardar-Parisi-Zhang equation in the weak-coupling regime. Physical Review A. 1992;45:7156–7161. 10.1103/PhysRevA.45.7156 9906788

[87] Masoudi AA, Khorrami M, Stastna M, Kohandel M. Dynamics of radial fractional growing surfaces. Europhysics Letters. 2012;100(1):16004 10.1209/0295-5075/100/16004

