# Supplementary figures and images for: Spatial patterns of dengue cases in Brazil
Source: PLoS One. 2017 Jul 17;12(7):e0180715. doi: 10.1371/journal.pone.0180715 (PMC5513438; doi:10.1371/journal.pone.0180715)

Latitude

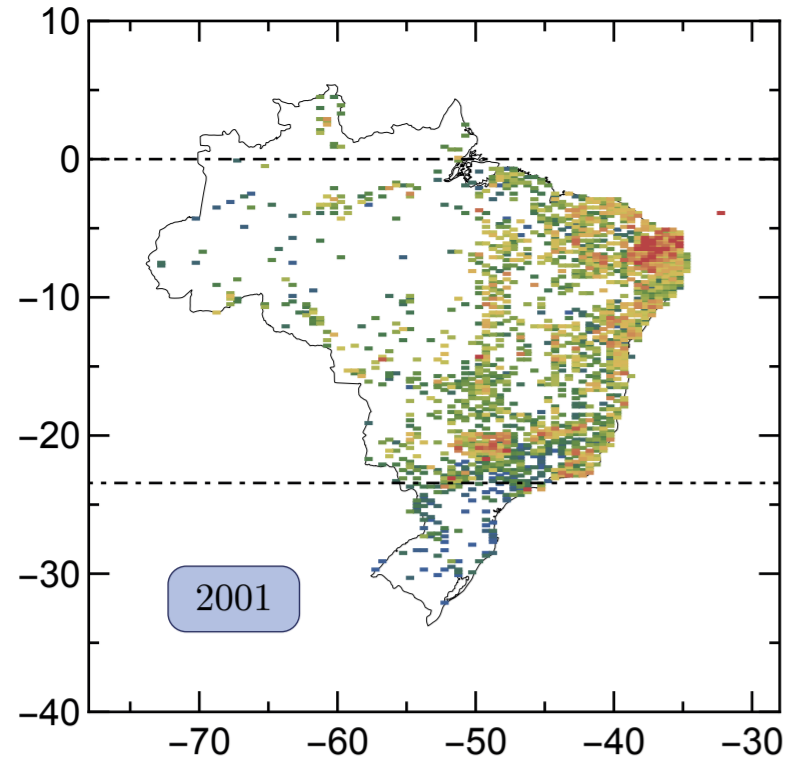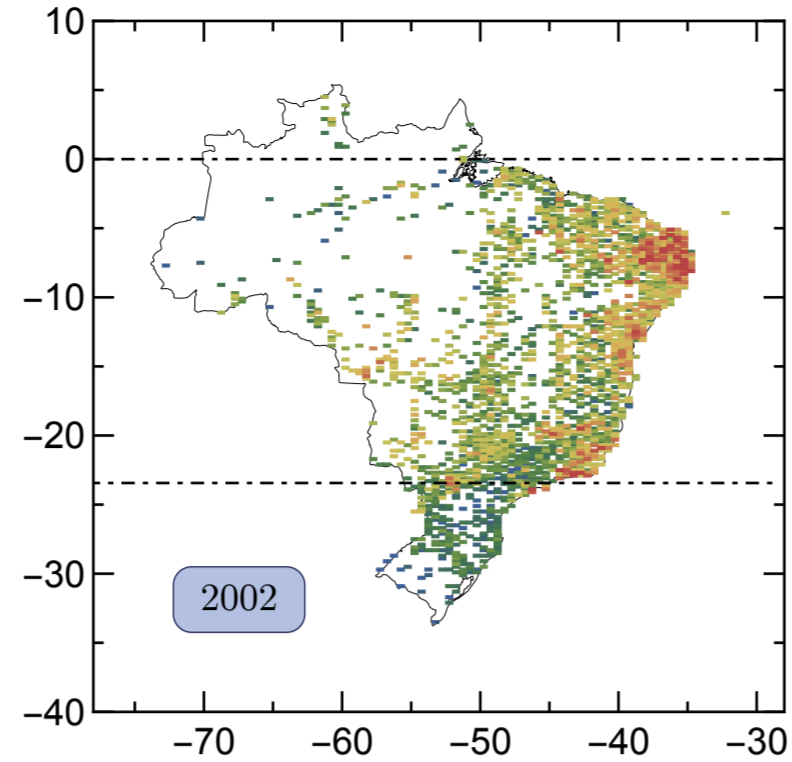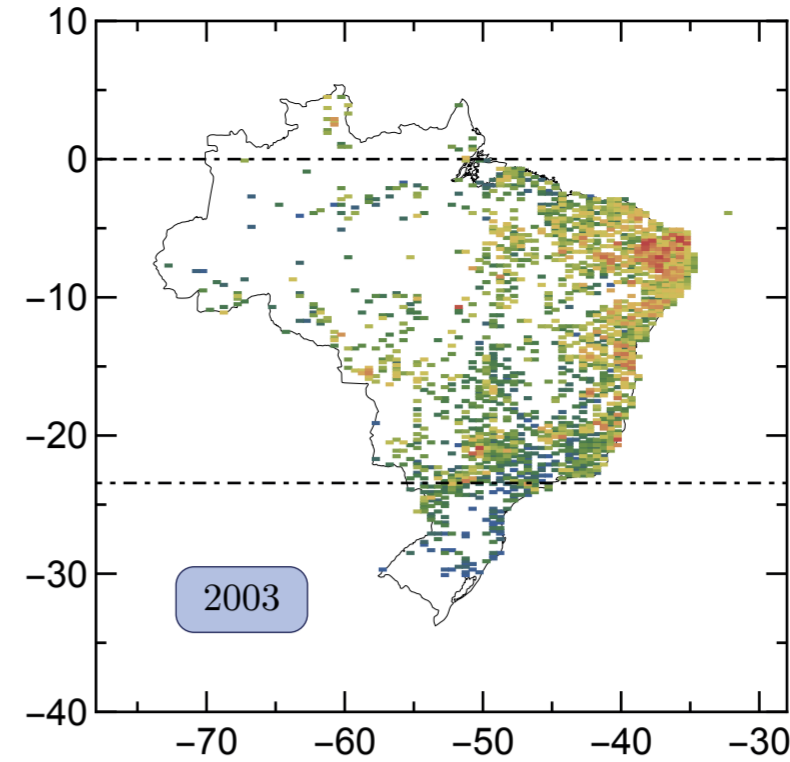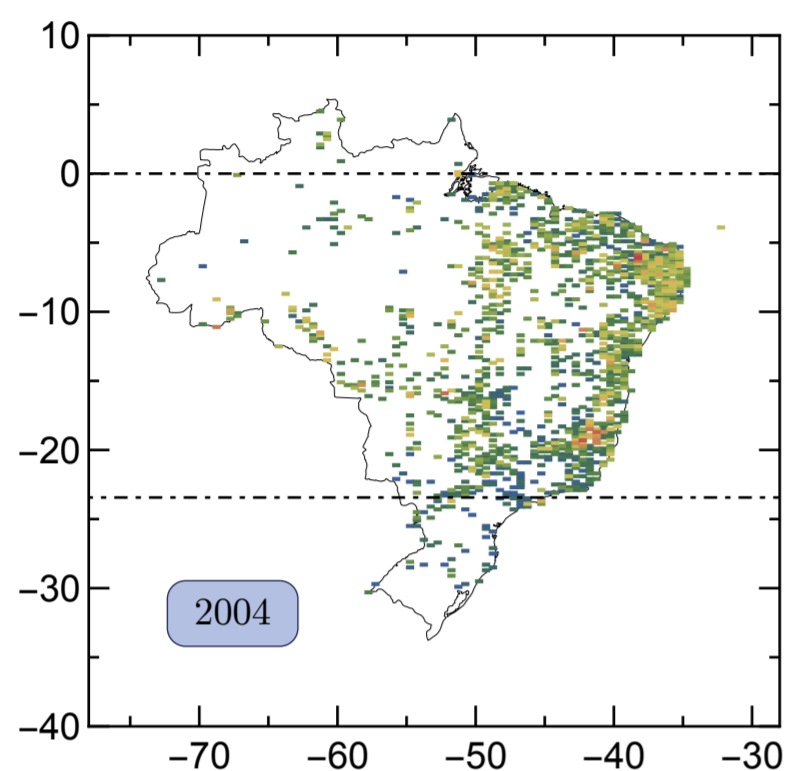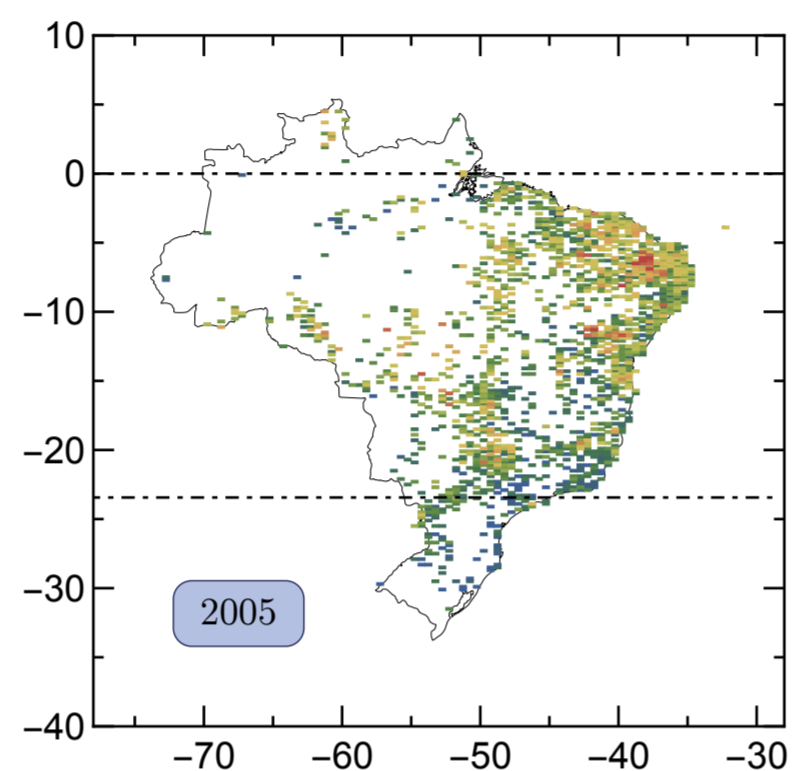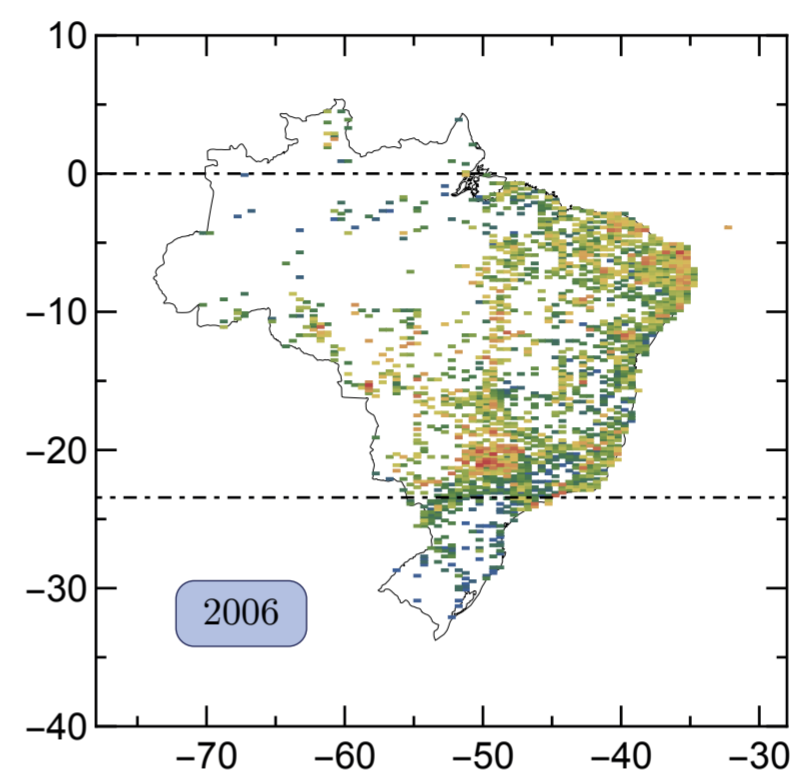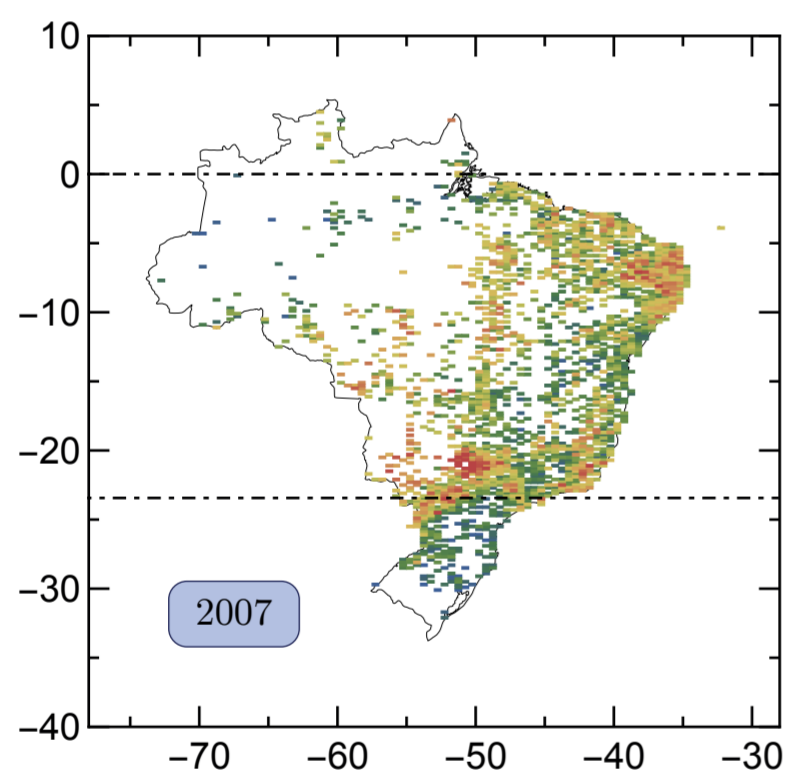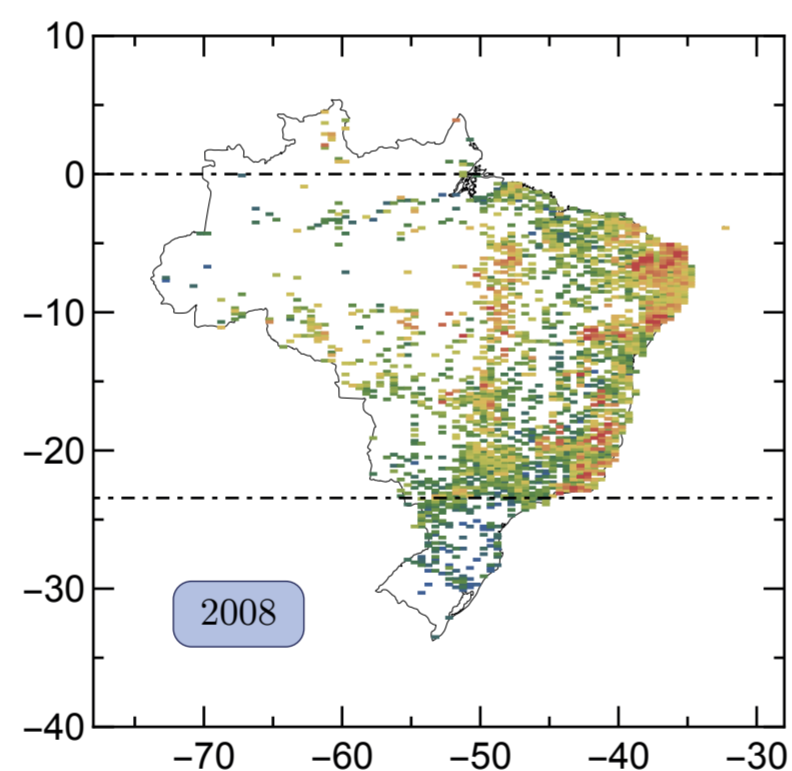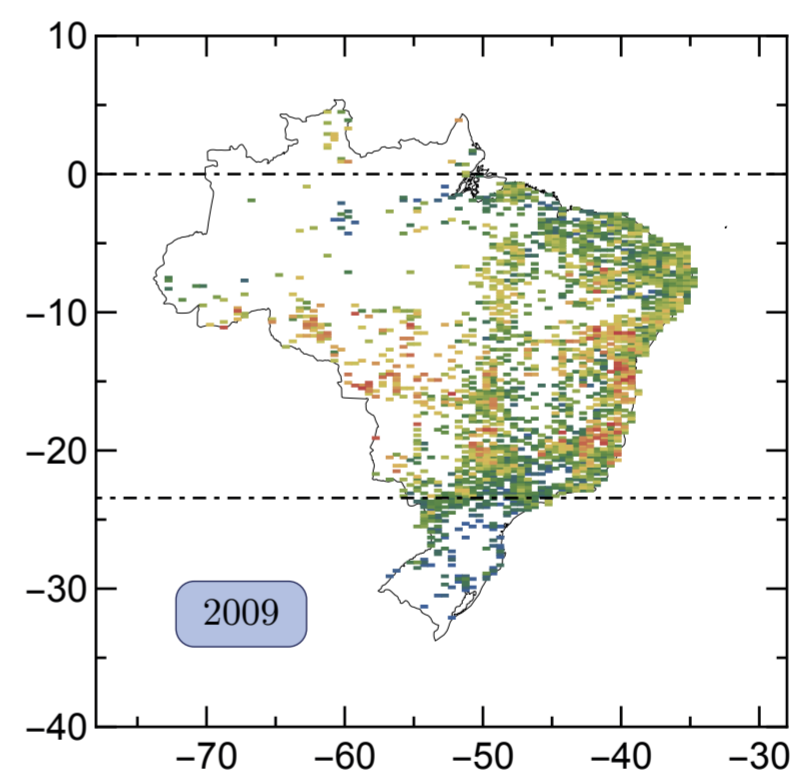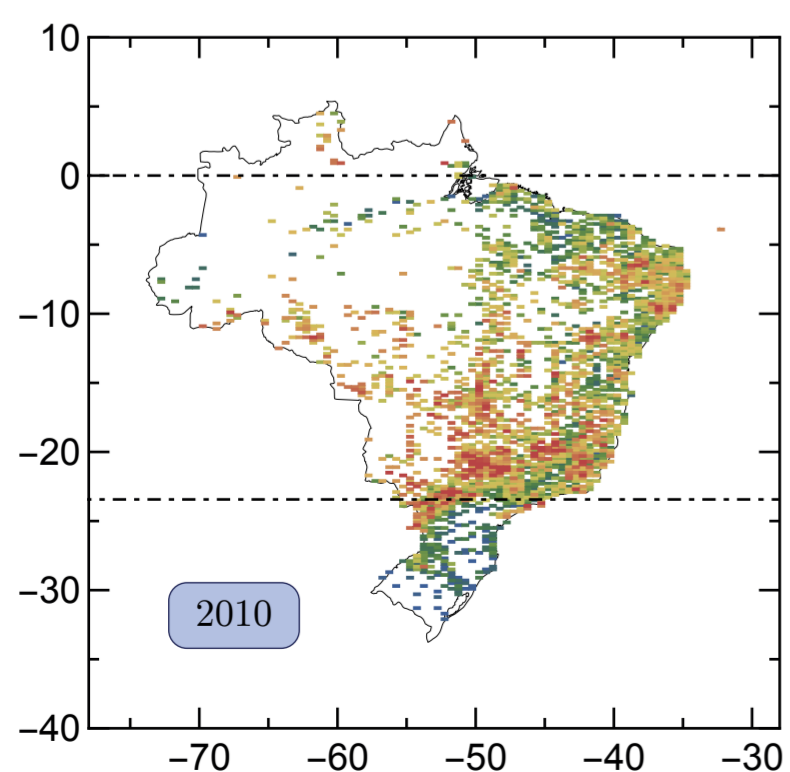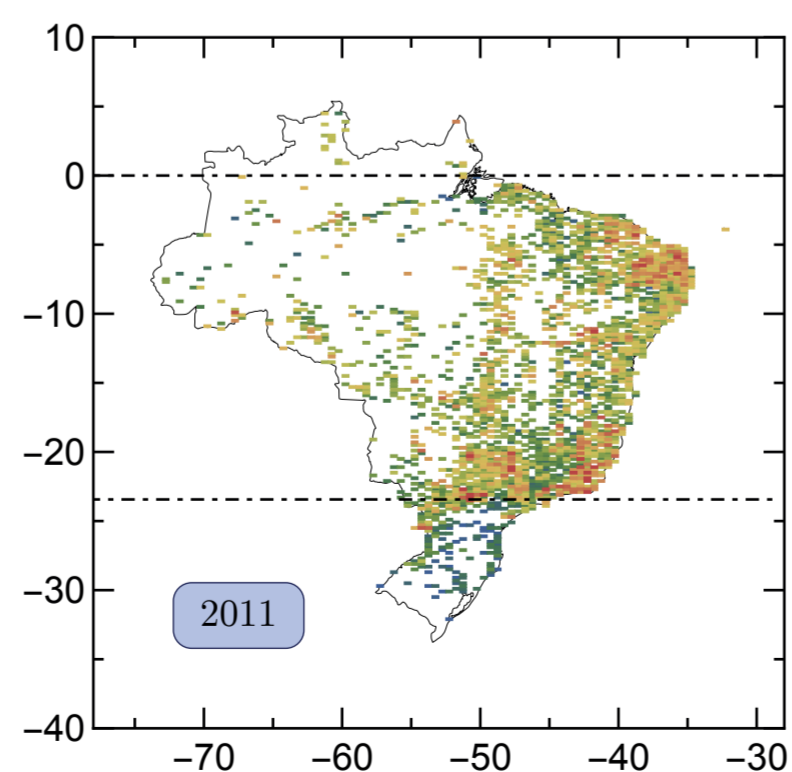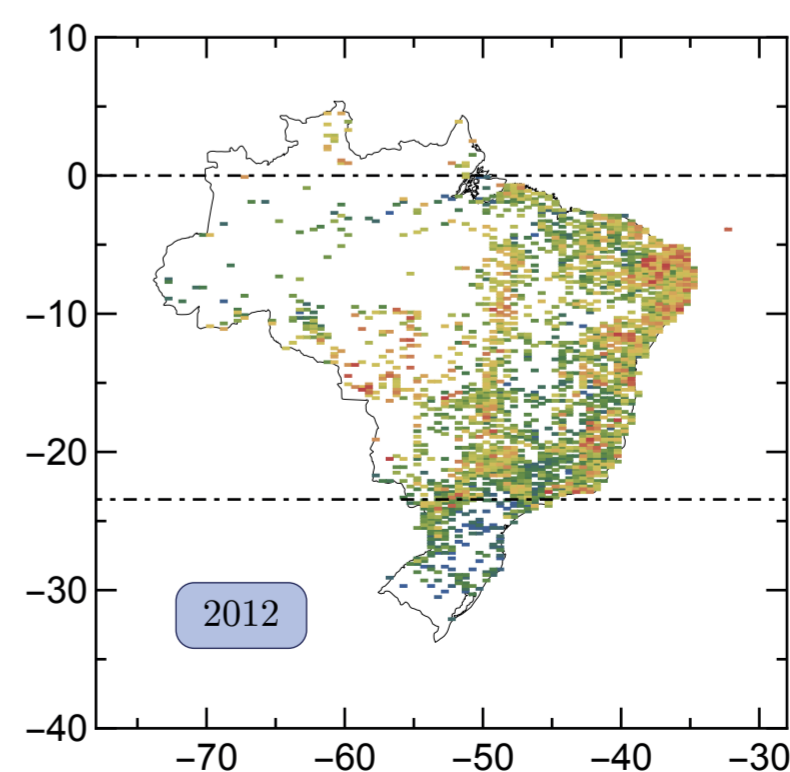

Longitude

Legenda

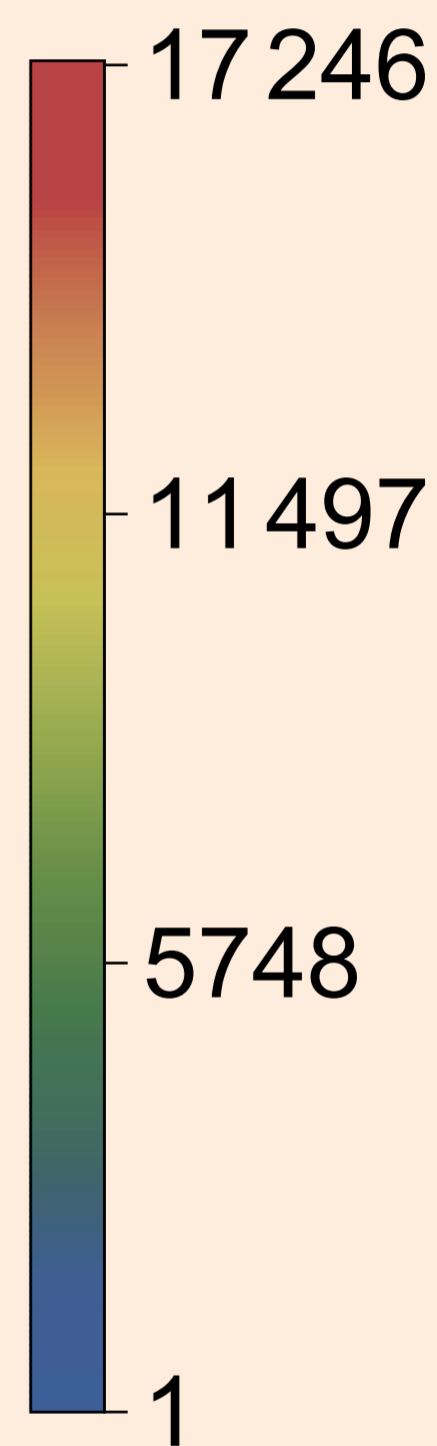

Supplement: S1 Fig — A general profile of the average number of dengue cases from 2001 up to 2012. Data on the population of the municipalities was projected for each year without census. The color scheme indicates that the major incidence of dengue is found in the extreme Northeastern and Southern Brazilian regions. The dashed lines correspond to the Equator and the Tropic of Capricorn. (PDF) [file pone.0180715.s002.pdf]
